# Genomic markers associated with successful treatment of hypertension with lisinopril: A pilot study 

**DOI:** 10.5414/CP203910

**Published:** 2021-03-26

**Authors:** Hania K. Flaten, Brandon J. Sonn, Jessica L. Saben, Shelby K. Shelton, John Schwartz, Karen Ryall, Andrew A. Monte

**Affiliations:** 1 Department of Emergency Medicine,; 2 Center for Bioinformatics & Personalized Medicine, University of Colorado School of Medicine,; 3 University of Colorado, Skaggs School of Pharmacy, and; 4 Rocky Mountain Poison and Drug Center, Denver Health and Hospital Authority, Denver, CO, USA

**Keywords:** ACE inhibitors, hypertension, lisinopril, pharmacogenomics, precision medicine

## Abstract

Objective: To identify single nucleotide variants (SNVs) associated with lisinopril effectiveness. Materials and methods: This was an observational study using a candidate gene approach to examine SNVs associated with lisinopril effectiveness. Drug effectiveness was defined as 10% decrease in systolic blood pressure at 1 week follow-up. We used the Illumina GWAS MEGA chip to examine variants in the renin/angiotensin pathway that may be associated with drug effectiveness. Results: 61 subjects were enrolled, and 33 (54.1%) were responsive to lisinopril therapy. SNVs in *AGT* (p = 0.0141), *REN* (p = 0.0192), and *ACE2* (p = 0.0002) were found to be associated with successful treatment on lisinopril. Conclusion and relevance: SNVs in the renin and angiotensin pathway are associated with lisinopril effectiveness in a pilot cohort of patients with uncontrolled hypertension.

**What is known about this subject**

- Only 50% of patients receiving antihypertensives achieve blood pressure control.
- Angiotensin converting enzyme inhibitors are the most commonly prescribed antihypertensive medication
- Interindividual genetic variants may contribute to drug effectiveness

**What this study adds**

- Genetic variants in AGT, REN, and ACE2 genes were associated with failure to achieve 10% systolic blood pressure decrease due to lisinopril.
- Larger studies are needed to determine if variants in the renin and angiotensin pathway affect lisinopril effectiveness.

## Introduction 

Hypertension (HTN) affects more than 1 billion people worldwide [1]. The Eighth Joint National Committee (JNC-8) guidelines suggest choosing HTN medications based on age, race, and comorbidities [2]. Despite the guidelines, 50% of patients with HTN fail to achieve target blood pressures on monotherapy resulting in more complex polypharmacy and repeat physician visits [2]. 

HTN is a complex, polygenic disease which may contribute to the shortcomings of conventional HTN treatment strategies. A novel approach to HTN therapy is to use genomic analyses to inform drug choices. Testing each patient for variants in drug targets, metabolic enzymes, and other genes involved with absorption, distribution, metabolism, and elimination allows the provider to select a drug based on an individual’s genomic profile. This could decrease adverse drug events, improve time to ideal HTN control, and result in fewer follow-up visits. Our objective was to identify single nucleotide variants (SNVs) associated with lisinopril effectiveness. 

## Materials and methods 

We evaluated and identified genetic markers associated with response to lisinopril, an angiotensin-converting enzyme inhibitor (ACEI). Our research is a pilot study, and secondary analysis of a prospective observational parent study (NCT02293096) which evaluated the pharmacogenomic effectiveness of metoprolol succinate. Patients with uncontrolled HTN, as defined by the JNC-8 guidelines, were referred from clinics and other clinical areas at the University of Colorado Hospital in Aurora, CO. Our secondary analysis included a subset of patients between 30 and 80 years of age started on lisinopril in addition to metoprolol succinate. Exclusion criteria included end-stage liver disease, glomerular filtration rate < 60 mL/min/1.73m^2^, pregnancy, American Association of Anesthesiologists (ASA) classification of > 3, prisoners or wards of the state, decisionally challenged individuals, heart rate < 60 beats per minute, AV block > 240 ms, active reactive airway disease, illicit drug use in the preceding 30 days (excluding marijuana), allergy to metoprolol succinate, severe peripheral arterial circulatory disorders, or patients with secondary causes of HTN. The study was approved by the Colorado Multiple Institutions Review Board and adheres to the Declaration of Helsinki. 

Enrolled patients were managed according to the JNC-8 HTN treatment guidelines. Patients were started on lisinopril 10 mg daily at the initial visit, and blood pressure was re-evaluated 1 week later. Those who responded to lisinopril were defined as individuals who had either a 10% decline in systolic blood pressure (SBP) or SBP < 140 at the week-1 visit. All others were considered non-responsive. 

**Figure 1.**
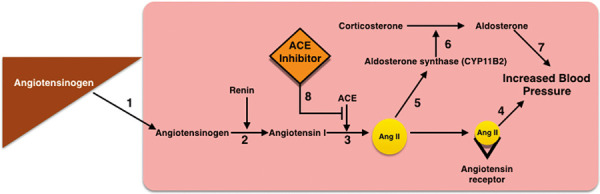
Angiotensin-converting enzyme (ACE) inhibitor interaction with the renin-angiotensin-aldosterone system. Angiotensinogen is released from the liver (#1) into the blood where it is converted to angiotensin I by renin (#2). Angiotensin I is converted to angiotensin II (ang II) by angiotensin converting enzyme (ACE, #3). Ang II binds angiotensin receptors which results in increased blood pressure (#4). Ang II also stimulates the production and release of aldosterone (#5, #6) which leads to increased blood pressure (#7). ACE inhibitors prevent the conversion of angiotensin I to ang II (#8).

The Ilumina MEGA chip (San Diego, CA, USA), which interrogates 1,696,817 SNVs was used for genetic analyses. Clinically relevant variants in the pathway for ACEI treatment (Figure 1) were included. Sequences were aligned to Genome Reference Consortium human genome version 37 (GRCh37). We first performed a candidate gene approach examining genes in the ACE pathway. A p-value of < 0.05 was used as a level of significance for the candidate gene approach. We then performed a genome-wide association study to examine additional variants that were not previously recognized. 

Data were analyzed using the R programming language. SNV association tests were performed with the SNVStats R package [3]. Association tests were performed using the Cochran-Armitage trend test with binary response to lisinopril as the outcome variable and race and ethnicity as covariates. SNVs with minor allele frequency (MAF) ≤ 1% and call rate across samples ≤ 80% were excluded from analysis. The biomaRt R package [4] was used to annotate SNVs with gene and phenotype information. 

## Results 

61 subjects with HTN were included in the analysis. 34 subjects were male (55.7%). The most common races and ethnicities included Caucasian (n = 33, 54.1%), African descent (n = 27, 44.3%), and Hispanic or Latino (n = 13, 21.3%). The mean age of patients enrolled in this study was 48.9 years (SD 9.2). 9 subjects (14.8%) reported a diagnosis of diabetes mellitus, and 11 (18.0%) reported current smoking. 

33 subjects (n = 54.1%) responded to lisinopril therapy. Mean starting SBP was similar between responders and non-responders (SBP 169 mmHg (SD 19.8) vs. 163 mmHg (SD 21.5), t = 0.87) although, as expected, the responders had significantly lower SBP at 1 week (SBP 139 mmHg (SD 20.7) vs. 160 mmHg (SD 23.7)), t = 0.0003*). Diastolic BPs (DBP) followed the same pattern (baseline DBP 95.4 mmHg (SD 9.7) vs. 94 mmHg (SD 19.9)). Genomic analyses revealed numerous SNVs associated with effectiveness of ACEI therapy in the targeted analysis (Table 1) and GWAS analysis (Online Supplemental material). 

## Discussion 

The identified SNVs have biologic relevance to ACEI response. Three SNVs (renin, angiotensinogen, and *ACE2*) were found in intronic regions of genes directly involved in the renin-angiotensin-aldosterone pathway. Intronic regions affect the rate of transcription, nuclear export, and transcript stability [5]. Thus, polymorphisms in intronic regions have potential to affect transcription and rates of expression. The angiotensinogen gene encodes angiotensinogen precursor. Mutations in this gene are associated with susceptibility to essential HTN [6]. Renin catalyzes the first step in the activation pathway of angiotensinogen, leading to aldosterone release, vasoconstriction, and increased blood pressure. In addition, renin cleaves angiotensinogen into angiotensin I. *ACE2* encodes angiotensin converting enzyme (ACE), which catalyzes the cleavage of angiotensin I into angiotensin II leading to vasoconstriction and increase in blood pressure (Figure 1). 

Other SNVs were found in genes associated with adverse events to ACEIs, such as *KCNIP4*, *MME*, *SLCO1B1*. Some *KCNIP4* variants induce problems regulating Kv4 channels leading to higher risk of adverse events attributable to ACEIs, as ACEIs prevent the breakdown of bradykinin. *MME* encodes neprilysin, which, along with ACE, degrades vasoactive peptides such as bradykinin and substance P. Patients taking ACEIs likely rely on neprilysin as a secondary mechanism to breakdown these vasoactive peptides. Therefore, variants in the *MME* gene may result in decreased function of neprilysin, leading to increased peptide mediators in the plasma in the presence of ACE inhibition. The accumulation of these peptides can result in cough and angioedema [7]. *SLCO1B1* encodes an organic anion transporter protein, OATP1B1, which is involved in hepatic drug clearance of several ACEIs [8]. Carriers of 521T>C variant showed increased plasma concentrations of enalapril. ACEIs are partly transported into bile and cleared from the plasma by the OATP1B1 transporter. 

While we are optimistic about our findings, there are several limitations of this work, most importantly the inclusion of only 61 total subjects and we determined initial effectiveness at the 1-week mark. It is possible that some patients may require longer duration of therapy or higher doses to reach maximal antihypertensive effects. Enrolled patients were predominantly of American Caucasian or African American descent. While the MEGA chip captures a wide range of ancestry variants, different ethnic populations will certainly have different frequencies of these SNVs and will likely have additional SNVs that may affect lisinopril effectiveness. Additionally, this study was only able to evaluate variants on the MEGA chip and is thus unable to assess association of other potential SNVs with lisinopril efficacy. These SNVs may indicate the genes involved in the pathway and regulation of blood pressure or they may represent type 1 errors in our analysis. 

Uncontrolled HTN can cause major health consequences, and we must rethink our approach to the optimal treatment of HTN. More studies must be conducted to determine the clinical utility of genotyping prior to anti-HTN prescription. Identifying patients who will not respond to ACEI therapy has potential to reduce healthcare costs. 

## Authors’ contributions 

HKF enrolled patients and drafted the manuscript. BJS performed the DNA extraction and genomic analyses. JLS provided laboratory support and aided in genomic analyses. SKS enrolled patients and provided edits on the manuscript. JS and KR performed the statistical analyses. AAM conceived the study, obtained funding for the study, and oversaw the work. 

## Availability of data and materials 

All data are available from the PI upon request. 

## Ethics approval 

This work was approved by the Colorado Multiple Institutional Review Board. All subjects provided written informed consent for inclusion and subsequent publication of these results. 

## Funding 

A.A.M. received support from the National Institutes of Health (NIH) K23 GM110516, NIH R35GM124939-01 and NIH CTSI UL1 TR001082. This project received seed grant funding from the American Medical Association Foundation. 

## Conflict of interest 

There is no conflict of interest for any author. 

**Table 1.** SNVs associated with renin-angiotensin-aldosterone system and metabolic pathways involved in ACE inhibitor metabolism.

SNV	p-value	Gene	Function
rs10864772	0.01	*AGT*	The protein encoded by this gene, pre-angiotensinogen or angiotensinogen precursor, is expressed in the liver and is cleaved by the enzyme renin in response to lowered blood pressure. The protein is involved in maintaining blood pressure and in the pathogenesis of essential hypertension. Mutations in this gene are associated with susceptibility to essential hypertension
rs11240695	0.02	*REN*	Renin catalyzes the first step in the activation pathway of angiotensinogen which leads to aldosterone release, vasoconstriction, and increase in blood pressure. Renin cleaves angiotensinogen to form angiotensin I, which is converted to angiotensin II by ACE, an important regulator of blood pressure.
rs4240157	< 0.01	*ACE2*	Angiotensin-converting enzyme family of dipeptidyl carboxydipeptidases. Catalyzes the cleavage of angiotensin I into angiotensin, and angiotensin II into the vasodilator angiotensin.
rs73107462	< 0.01	*KCNIP4*	KCNIP4 regulates Kv4 potassium channels. Mutation in *KCNIP4* gene may lead to stimulation of sensory nerve afferents within the lung from accumulation of bradykinin.
rs17520919	< 0.01		
rs358826	0 < 0.01		
rs1398831	< 0.01		
rs6838195	< 0.01		
rs10000092	0.01		
rs56807277	0.02		
rs16869949	0.01		
rs1352798	0.02		
rs60028998	0.02		
rs16870400	0.03		
rs7683672	0.03		
rs9991631	0.03		
rs2137650	0.03		
rs6850480	0.03		
rs73801665	0.03		
rs16870831	0.03		
rs1495522	0.04		
rs11734403	0.05		
rs114695525	0.05		
rs7649252	0.01	*MME*	*MME* encodes neprilysin which breaks down bradykinin along with ACE. Neprilysin along with ACE degrades vasoactive peptides such as bradykinin and substance P.
rs9853221	0.02		
rs4149063	0.04	*SLCO1B1*	SLCO1B1 encodes an organic anion transporter protein, OATP1B1, which is involved in hepatic drug clearance of enalapril. Both ACEIs and ARBs [8] are partly transported into bile and cleared from the plasma by the OATP1B1 transporter.

SNV = single nucleotide variant; ACE = angiotensin-converting enzyme; ARB = angiotensin receptor blocker.

## Supplemental material

Supplemental material SNVs from Ilumina MEGA with significant finding p-values (p < 0.05).
