# Supplementary material for: Genomic markers associated with successful treatment of hypertension with lisinopril: A pilot study
Source: Int J Clin Pharmacol Ther. 2021 Mar 26;59(7):506–10. doi: 10.5414/CP203910 (PMC8383846; doi:10.5414/CP203910)
Supplement: Supplemental material [file intjclinpharmacol-59-506-S01.pdf]

Online Supplemental material. SNVs from Ilumina MEGA with significant finding p-values ($p < 0.05$). Blank space in HGNC-Symbol indicates no gene information available in the Biomart database for that SNV.

**refSNV_id**	**p-value**	**hgnc_symbol (if available)**
rs1360485	1.16E-05	HMGB1
rs79376117	1.23E-05	
rs3786829	2.21E-05	RYR1
rs10242381	3.00E-05	
rs981101	3.08E-05	
rs72879030	3.15E-05	
rs55765108	3.37E-05	
rs2469518	3.57E-05	KCNQ3
rs73730455	3.92E-05	
rs1529770	4.09E-05	
rs1331817	4.27E-05	
rs76990086	5.12E-05	GRIN2A
rs3213822	5.13E-05	PDZRN3
rs6953995	5.50E-05	
rs249048	6.47E-05	
rs10020323	6.51E-05	NWD2
rs1882247	6.78E-05	
rs73591018	6.95E-05	
rs2000631	7.03E-05	
rs6725924	8.15E-05	PID1
rs6473516	8.27E-05	
rs114349755	8.38E-05	
rs72896671	8.38E-05	
rs13392981	8.68E-05	
rs1714524	8.74E-05	
rs2553825	8.97E-05	
rs56897154	9.10E-05	ZNF536
rs394750	9.15E-05	SCAI
rs10782722	9.75E-05	
rs2215298	9.80E-05	
rs10462005	1.03E-04	
rs10871427	1.03E-04	
rs12414094	1.04E-04	
rs11157080	1.16E-04	
rs1424065	1.22E-04	
rs7618619	1.23E-04	
rs57778082	1.24E-04	PTPRN2
rs7720260	1.26E-04	ABLIM3
rs1865350	1.27E-04	
rs7329753	1.27E-04	

rs12712962	1.28E-04	PRKCE
rs11770659	1.34E-04	
rs608895	1.42E-04	SCAI
rs6876947	1.43E-04	
rs9968111	1.45E-04	PLD1
rs2875745	1.50E-04	
rs3898665	1.70E-04	
rs666812	1.71E-04	TEK
rs7914342	1.76E-04	
rs17139341	1.87E-04	
rs9482193	1.90E-04	
rs10833965	1.93E-04	
rs11846873	1.94E-04	
rs59776579	1.95E-04	
rs6586371	2.07E-04	SLC35F3
rs1773241	2.11E-04	
rs4851333	2.12E-04	
rs4389183	2.14E-04	CA10
rs6932730	2.17E-04	
rs4684185	2.18E-04	
rs2850488	2.29E-04	
rs1316505	2.34E-04	
rs4240157	2.36E-04	ACE2
rs7176947	2.36E-04	
rs17015816	2.44E-04	CNTN4
rs6494119	2.44E-04	
rs10158644	2.47E-04	
rs998587	2.56E-04	
rs10071604	2.56E-04	SLIT3
rs11858750	2.58E-04	OTUD7A
rs3741303	2.58E-04	NNMT
rs10158460	2.58E-04	
rs241232	2.66E-04	
rs10211247	2.67E-04	
rs74754799	2.73E-04	PTEN
rs12305013	0.020758	ETV6
rs11054481	0.022604	ETV6
rs7953505	0.025527	ETV6
rs73052077	0.034588	ETV6
rs10491993	0.034655	ETV6
rs2515767	0.043267	ETV6
rs76803639	0.047152	ETV6
rs12263457	0.028802	PRKCQ
